# Supplementary material for: Antibiotic-induced acceleration of type 1 diabetes alters maturation of innate intestinal immunity
Source: eLife. 2018 Jul 25;7:e37816. doi: 10.7554/eLife.37816 (PMC6085123; doi:10.7554/eLife.37816)
Supplement: Supplementary file 1. [file elife-37816-supp1.docx]

| **Supplementary File 1.** **Microbial metabolic pathways significantly differentiated by 1PAT in male (M) and female (F) mice at P21 and P49, based on metagenomic analysis.**  **[ +, up-regulated by 1PAT; -, down-regulated by 1PAT; M: Male; F: Female]** | | | | |
| --- | --- | --- | --- | --- |
| **Microbial pathway** | **M:P21** | **M:P49** | **F:P21** | **F:P49** |
| P4−PWY: superpathway of L−lysine, L−threonine and L−methionine biosynthesis I | **+** | **+** | **+** | **+** |
| PWY0−781: aspartate superpathway | **+** | **+** | **+** | **+** |
| NAGLIPASYN−PWY: lipid IVA biosynthesis | **+** | **+** | **+** |  |
| DENOVOPURINE2−PWY: superpathway of purine nucleotides de novo biosynthesis II | **+** | **+** |  | **+** |
| P108−PWY: pyruvate fermentation to propanoate I | **+** | **+** |  | **+** |
| PHOSLIPSYN−PWY: superpathway of phospholipid biosynthesis I (bacteria) | **+** | **+** |  | **+** |
| PWY−5345: superpathway of L−methionine biosynthesis (by sulfhydrylation) | **+** | **+** |  | **+** |
| PWY−5695: urate biosynthesis/inosine 5'−phosphate degradation | **+** | **+** |  | **+** |
| PWY−7187: pyrimidine deoxyribonucleotides de novo biosynthesis II | **+** | **+** |  | **+** |
| PWY−7211: superpathway of pyrimidine deoxyribonucleotides de novo biosynthesis | **+** | **+** |  | **+** |
| PWY66−409: superpathway of purine nucleotide salvage | **+** | **+** |  | **+** |
| SER−GLYSYN−PWY: superpathway of L−serine and glycine biosynthesis I | **+** | **+** |  | **+** |
| SULFATE−CYS−PWY: superpathway of sulfate assimilation and cysteine biosynthesis | **+** | **+** |  | **+** |
| HOMOSER−METSYN−PWY: L−methionine biosynthesis I | **+** |  | **+** | **+** |
| MET−SAM−PWY: superpathway of S−adenosyl−L−methionine biosynthesis | **+** |  | **+** | **+** |
| METSYN−PWY: L−homoserine and L−methionine biosynthesis | **+** |  | **+** | **+** |
| PWY−5347: superpathway of L−methionine biosynthesis (transsulfuration) | **+** |  | **+** | **+** |
| PWY−7237: myo−, chiro− and scillo−inositol degradation | **+** |  | **+** | **+** |
| RHAMCAT−PWY: L−rhamnose degradation I | **+** |  | **+** | **+** |
| FASYN−ELONG−PWY: fatty acid elongation – saturated | **+** | **+** |  |  |
| GLYCOLYSIS−E−D: superpathway of glycolysis and Entner−Doudoroff | **+** | **+** |  |  |
| HEXITOLDEGSUPER−PWY: superpathway of hexitol degradation (bacteria) | **+** | **+** |  |  |
| P461−PWY: hexitol fermentation to lactate, formate, ethanol and acetate | **+** | **+** |  |  |
| PWY−6628: superpathway of L−phenylalanine biosynthesis | **+** | **+** |  |  |
| PWY−7664: oleate biosynthesis IV (anaerobic) | **+** | **+** |  |  |
| PWY0−1061: superpathway of L−alanine biosynthesis | **+** | **+** |  |  |
| PWY0−862: (5Z)−dodec−5−enoate biosynthesis | **+** | **+** |  |  |
| ARGSYNBSUB−PWY: L−arginine biosynthesis II (acetyl cycle) | **+** |  |  | **+** |
| CALVIN−PWY: Calvin−Benson−Bassham cycle | **+** |  |  | **+** |
| DAPLYSINESYN−PWY: L−lysine biosynthesis I | **+** |  |  | **+** |
| PWY−5659: GDP−mannose biosynthesis | **+** |  |  | **+** |
| PWY−821: superpathway of sulfur amino acid biosynthesis (Saccharomyces cerevisiae) | **+** |  |  | **+** |
| PWY0−845: superpathway of pyridoxal 5'−phosphate biosynthesis and salvage | **+** |  |  | **+** |
| PYRIDOXSYN−PWY: pyridoxal 5'−phosphate biosynthesis I | **+** |  |  | **+** |
| ASPASN−PWY: superpathway of L−aspartate and L−asparagine biosynthesis |  | **+** |  | **+** |
| HISDEG−PWY: L−histidine degradation I |  | **+** |  | **+** |
| P441−PWY: superpathway of N−acetylneuraminate degradation |  | **+** |  | **+** |
| PWY−6168: flavin biosynthesis III (fungi) |  | **+** |  | **+** |
| PWY−7383: anaerobic energy metabolism (invertebrates, cytosol) |  | **+** |  | **+** |
| PWY0−1298: superpathway of pyrimidine deoxyribonucleosides degradation |  | **+** |  | **+** |
| P125−PWY: superpathway of (R,R)−butanediol biosynthesis | **+** |  |  |  |
| P161−PWY: acetylene degradation | **+** |  |  |  |
| PWY−6901: superpathway of glucose and xylose degradation | **+** |  |  |  |
| PWY4FS−7: phosphatidylglycerol biosynthesis I (plastidic) | **+** |  |  |  |
| PWY4FS−8: phosphatidylglycerol biosynthesis II (non−plastidic) | **+** |  |  |  |
| CALVIN−PWY: Calvin−Benson−Bassham cycle |  | **+** |  |  |
| PWY−6897: thiamin salvage II |  | **+** |  |  |
| PWY−7184: pyrimidine deoxyribonucleotides de novo biosynthesis I |  | **+** |  |  |
| PWY0−166: superpathway of pyrimidine deoxyribonucleotides de novo biosynthesis (E. coli) |  | **+** |  |  |
| ARGSYN−PWY: L−arginine biosynthesis I (via L−ornithine) |  |  | **+** |  |
| ARO−PWY: chorismate biosynthesis I |  |  | **+** |  |
| COMPLETE−ARO−PWY: superpathway of aromatic amino acid biosynthesis |  |  | **+** |  |
| DAPLYSINESYN−PWY: L−lysine biosynthesis I |  |  | **+** |  |
| GLUTORN−PWY: L−ornithine biosynthesis |  |  | **+** |  |
| PWY−6122: 5−aminoimidazole ribonucleotide biosynthesis II |  |  | **+** |  |
| PWY−6163: chorismate biosynthesis from 3−dehydroquinate |  |  | **+** |  |
| PWY−6277: superpathway of 5−aminoimidazole ribonucleotide biosynthesis |  |  | **+** |  |
| PWY−7400: L−arginine biosynthesis IV (archaebacteria) |  |  | **+** |  |
| PWY0−1261: anhydromuropeptides recycling |  |  | **+** |  |
| FERMENTATION−PWY: mixed acid fermentation |  |  |  | **+** |
| NONOXIPENT−PWY: pentose phosphate pathway (non−oxidative branch) |  |  |  | **+** |
| PWY−6270: isoprene biosynthesis I |  |  |  | **+** |
| PWY−6353: purine nucleotides degradation II (aerobic) |  |  |  | **+** |
| PWY−6595: superpathway of guanosine nucleotides degradation (plants) |  |  |  | **+** |
| PWY−6608: guanosine nucleotides degradation III |  |  |  | **+** |
| PWY0−162: superpathway of pyrimidine ribonucleotides de novo biosynthesis |  |  |  | **+** |
| PYRIDNUCSYN−PWY: NAD biosynthesis I (from aspartate) |  |  |  | **+** |
| SALVADEHYPOX−PWY: adenosine nucleotides degradation II |  |  |  | **+** |
| COA−PWY−1: coenzyme A biosynthesis II (mammalian) |  | **-** |  |  |
| PWY−6527: stachyose degradation |  | **-** |  |  |
| UDPNAGSYN−PWY: UDP−N−acetyl−D−glucosamine biosynthesis I |  |  | **-** |  |
