# Supplementary material for: Antibiotic-induced acceleration of type 1 diabetes alters maturation of innate intestinal immunity
Source: eLife. 2018 Jul 25;7:e37816. doi: 10.7554/eLife.37816 (PMC6085123; doi:10.7554/eLife.37816)
Supplement: Supplementary file 2. [file elife-37816-supp2.docx]

| **Metabolite** | **VIP** | **Fold change** | **p-value** | **Groups** |  |
| --- | --- | --- | --- | --- | --- |
| **Serum** |  |  |  |  |  |
| P15 |  |  |  |  |  |
| 1,5-anhydroglucitol | 1.5 | 4.9 | 0.01 | All (males plus females)** |  |
| alanine | 1.5 | 1.8 | 0.01 | All |  |
| phenylalanine | 1.5 | 1.7 | 0.01 | All |  |
| citric acid | 1.4 | 1.9 | 0.01 | All |  |
| glutamine | 1.4 | 2.2 | 0.03 | All |  |
| valine | 1.4 | 1.6 | 0.02 | All |  |
| alpha-ketoglutarate | 1.3 | 1.5 | 0.02 | All |  |
| epsilon caprolactam | 1.3 | 1.7 | 0.03 | All |  |
| glucose | 1.3 | 35.7 | 0.04 | All |  |
| isoleucine | 1.2 | 1.4 | 0.04 | All |  |
| lysine | 1.2 | 1.3 | 0.04 | All |  |
| glycerol alpha phosphate | 1.5 | -1.6 | 0.01 | All |  |
| capric acid | 1.4 | -1.9 | 0.01 | All |  |
| lauric acid | 1.4 | -1.9 | 0.02 | All |  |
| phenylacetic acid | 1.4 | -1.7 | 0.02 | All |  |
| catechol likely | 1.3 | -1.5 | 0.05 | All |  |
| myristic acid | 1.3 | -1.7 | 0.04 | All |  |
| putrescine | 1.3 | -1.6 | 0.04 | All |  |
| P23 |  |  |  |  |  |
| pipecolinic acid | 1.9 | -1.4 | 0.01 | All |  |
| alanine | 1.8 | -1.6 | 0.02 | All |  |
| phenylalanine | 1.7 | -1.6 | 0.01 | All |  |
| succinic acid | 1.7 | -2.6 | 0.01 | All |  |
| glutamine | 1.6 | -3.9 | 0.01 | All |  |
| fumaric acid | 1.5 | -1.4 | 0.05 | All |  |
| phthalic acid | 1.3 | -1.7 | 0.04 | All |  |
| P42 |  |  |  |  |  |
| uracil | 1.8; 1.8; 1.6 | 2.6; 2.4; 3.0 | <0.01; 0.02; 0.04 | All; Male; Female |  |
| 2-hydroxybutanoic acid | 1.7 | 2.0 | 0.02 | Female |  |
| creatine major dehydrated | 1.6 | 1.5 | 0.03 | Female |  |
| glycerol alpha phosphate | 1.6 | 1.5 | 0.03 | Male |  |
| enolpyruvate nist | 1.5 | 1.5 | 0.05 | Female |  |
| n-methylalanine | 1.0 | 1.4 | 0.05 | All |  |
| lysine | 1.6 | -1.4 | 0.04 | Female |  |
| palmitic acid | 1.6 | -1.1 | 0.04 | Female |  |
| octadecanol | 1.6; 1.6 | -1.3; -1.4 | 0.02; 0.03 | All, Male |  |
| 1-monopalmitin | 1.4 | -1.3 | 0.03 | All |  |
| glucose | 1.4; 1.4 | -1.9; -2.2 | 0.05; 0.05 | All; Male |  |
| lauric acid | 1.6; 1.6 | -1.3; -1.4 | 0.02; 0.02 | All; Male |  |
| **Liver** |  |  |  |  |  |
| P23 |  |  |  |  |  |
| citric acid | 1.5 | 4.0 | 0.02 | All |  |
| uridine | 1.7 | -2.4 | 0.01 | All |  |
| tyrosine | 1.6 | -1.5 | 0.02 | All |  |
| isoleucine | 1.6 | -1.3 | 0.01 | All |  |
| arabitol | 1.5 | -1.9 | 0.03 | All |  |
| valine | 1.5 | -1.3 | 0.02 | All |  |
| sophorose | 1.4 | -1.7 | 0.04 | All |  |
| P42 |  |  |  |  |  |
| isoleucine | 1.9; 1.6; 1.8 | 1.5; 1.5; 1.5 | <0.01; 0.04; 0.03 | All; Male; Female |  |
| uracil | 1.7; 1.7 | 1.7, 2.5 | <0.01, 0.02 | All; Female |  |
| methionine | 1.6 | 1.6 | 0.03 | All |  |
| phenylalanine | 1.5; 1.5 | 1.7; 1.6 | 0.03; 0.04 | All; Female |  |
| valine | 1.7 | -1.5 | 0.02 | All |  |
| glucose 6 phosphate | 1.4 | -3.2 | 0.04 | Female |  |
| dioctylphtalate | 1.0 | -1.4 | 0.05 | Male |  |
| *The metabolites observed as significant in more than one time point or that were both significant in serum and liver in the same direction were highlighted.  ** Data are presented for males plus females (all) exposed to 1PAT vs Control (male plus female); ‘Male’ indicates significant results obtained from male 1PAT vs male control; ‘Female’ indicates significant results obtained from female 1PAT vs female control. | | | | | |

**Supplementary File 2. Metabolites differentially regulated by 1PAT in serum and liver of NOD mice early in life.**
