# Supplementary material for: Antibiotic-induced acceleration of type 1 diabetes alters maturation of innate intestinal immunity
Source: eLife. 2018 Jul 25;7:e37816. doi: 10.7554/eLife.37816 (PMC6085123; doi:10.7554/eLife.37816)
Supplement: Supplementary file 6. [file elife-37816-supp6.docx]

**Supplementary File 6.**

**Supplementary File Table 1a. Ileal immune genes significantly differentially expressed between 1PAT and control in P23 male NOD mice.**

| **Gene** | **Description** | **log2FoldChange** | **p-value** | **p-adjusted** |
| --- | --- | --- | --- | --- |
| *Btla* | B and T lymphocyte associated | 1.45 | 1.08E-05 | 3.44E-04 |
| *C2* | complement component 2 (within H-2S) | 1.93 | 3.63E-04 | 9.38E-03 |
| *C3* | complement component 3 | 1.63 | 5.58E-06 | 2.01E-04 |
| *Ccl19* | chemokine (C-C motif) ligand 19 | 1.93 | 1.85E-08 | 1.25E-06 |
| *Ccl2* | chemokine (C-C motif) ligand 2 | -1.43 | 1.07E-03 | 2.14E-02 |
| *Ccr4* | chemokine (C-C motif) receptor 4 | -2.00 | 3.41E-04 | 9.38E-03 |
| *Ccr6* | chemokine (C-C motif) receptor 6 | 1.50 | 1.84E-06 | 7.11E-05 |
| *Ccr7* | chemokine (C-C motif) receptor 7 | 1.45 | 7.05E-05 | 2.12E-03 |
| *Cd19* | CD19 antigen | 3.08 | 2.67E-20 | 7.24E-18 |
| *Cd2* | CD2 antigen | 1.25 | 4.16E-04 | 1.02E-02 |
| *Cd22* | CD22 antigen | 1.14 | 6.71E-04 | 1.45E-02 |
| *Cd79b* | CD79B antigen | 2.10 | 2.20E-10 | 2.39E-08 |
| *Cd83* | CD83 antigen | 1.39 | 1.01E-05 | 3.43E-04 |
| *Clu* | clusterin | 2.80 | 9.11E-21 | 4.94E-18 |
| *Cxcl13* | chemokine (C-X-C motif) ligand 13 | 1.72 | 7.97E-07 | 3.93E-05 |
| *Cxcl9* | chemokine (C-X-C motif) ligand 9 | -1.66 | 4.69E-04 | 1.06E-02 |
| *Ddx58* | DEAD (Asp-Glu-Ala-Asp) box polypeptide 58 | 1.27 | 4.65E-04 | 1.06E-02 |
| *Gzmb* | granzyme B | -2.01 | 1.70E-06 | 7.09E-05 |
| *H2-DMb2* | histocompatibility 2, class II, locus Mb2 | 1.89 | 2.00E-09 | 1.55E-07 |
| *Il17f* | interleukin 17F | -1.27 | 2.40E-03 | 4.07E-02 |
| *Il18* | interleukin 18 | -2.70 | 5.55E-15 | 7.52E-13 |
| *Irf7* | interferon regulatory factor 7 | 1.58 | 2.31E-03 | 4.03E-02 |
| *Lef1* | lymphoid enhancer binding factor 1 | 1.52 | 3.52E-04 | 9.38E-03 |
| *Ltb* | lymphotoxin B | 1.94 | 6.66E-10 | 6.02E-08 |
| *Ms4a1* | membrane-spanning 4-domains, subfamily A, member 1 | 2.59 | 2.01E-16 | 3.63E-14 |
| *Msr1* | macrophage scavenger receptor 1 | -1.05 | 1.38E-03 | 2.57E-02 |
| *Nfil3* | nuclear factor, interleukin 3, regulated | -1.09 | 2.14E-03 | 3.86E-02 |
| *Pax5* | paired box 5 | 2.16 | 3.56E-07 | 1.93E-05 |
| *Pla2g2a* | phospholipase A2, group IIA (platelets, synovial fluid) | -1.03 | 1.35E-03 | 2.57E-02 |
| *Socs3* | suppressor of cytokine signaling 3 | -2.55 | 1.52E-06 | 6.88E-05 |
| *Tmem173* | transmembrane protein 173 | -1.52 | 7.51E-04 | 1.57E-02 |
| *Tnfrsf13c* | tumor necrosis factor receptor superfamily, member 13c | 2.38 | 6.02E-08 | 3.62E-06 |

**Supplementary File Table 1b. Ileal immune genes significantly different in expression between 1PAT and control in P23 female NOD mice.**

| **Gene** | **Description** | **log2FoldChange** | **p-value** | **p-adjusted** |
| --- | --- | --- | --- | --- |
| *Cxcl9* | chemokine (C-X-C motif) ligand 9 | -1.41 | 1.17E-06 | 1.10E-04 |
| *Bcl3* | B cell leukemia/lymphoma 3 | -1.20 | 1.01E-05 | 5.96E-04 |
| *Socs1* | suppressor of cytokine signaling 1 | -1.11 | 7.43E-04 | 2.92E-02 |
| *Nt5e* | 5' nucleotidase, ecto | 1.84 | 5.56E-07 | 6.55E-05 |
| *Cfd* | complement factor D (adipsin) | 1.18 | 1.78E-03 | 4.93E-02 |
| *Pla2g2a* | phospholipase A2, group IIA (platelets, synovial fluid) | -0.82 | 2.06E-04 | 8.81E-03 |
| *Il22* | interleukin 22 | -1.92 | 1.99E-06 | 1.56E-04 |
| *Tap1* | transporter 1, ATP-binding cassette, sub-family B (MDR/TAP) | -0.96 | 8.52E-04 | 3.01E-02 |
| *Tmem173* | transmembrane protein 173 | -1.14 | 1.22E-05 | 6.41E-04 |
| *Socs3* | suppressor of cytokine signaling 3 | -2.68 | 7.68E-24 | 3.62E-21 |
| *Blnk* | B cell linker | -0.88 | 1.23E-03 | 3.78E-02 |
| *Cxcl10* | chemokine (C-X-C motif) ligand 10 | -1.40 | 2.01E-05 | 9.44E-04 |
| *Abcb1a* | ATP-binding cassette, sub-family B (MDR/TAP), member 1A | 1.68 | 3.89E-08 | 6.11E-06 |
| *Il18* | interleukin 18 | -1.58 | 4.55E-06 | 3.06E-04 |
| *Nos2* | nitric oxide synthase 2, inducible | -2.27 | 5.77E-11 | 1.36E-08 |
| *Psmb9* | proteasome (prosome, macropain) subunit, beta type 9 (large multifunctional peptidase 2) | -0.92 | 8.94E-04 | 3.01E-02 |
| *Ccl2* | chemokine (C-C motif) ligand 2 | -1.06 | 1.28E-03 | 3.78E-02 |
