# Supplementary material for: Antibiotic-induced acceleration of type 1 diabetes alters maturation of innate intestinal immunity
Source: eLife. 2018 Jul 25;7:e37816. doi: 10.7554/eLife.37816 (PMC6085123; doi:10.7554/eLife.37816)
Supplement: Supplementary file 7. [file elife-37816-supp7.docx]

**List of primer sequences used for RT-qPCR analysis.**

| **Target** | **Primer** | **Sequence** |
| --- | --- | --- |
| 18S | F | CATTCGAACGTCTGCCCTAT |
|  | R | CCTCCAATGGATCCTCGTTA |
| *Hprt* | F | TCAGTCAACGGGGGACATAAA |
|  | R | GGGGCTGTACTGCTTAACCAG |
| *Saa1/2* | F | GGTTTTTTTCATTTGTTCAC |
|  | R | TCTCTTCCATCACTGATTTTCT |
| *Saa3* | F | AAGAAGCTGGTCAAGGGTCTAG |
|  | R | TCTCTGGCATCGCTGATGACTT |
| *Nos2* | F | GTTCTCAGCCCAACAATACAAGA |
|  | R | GTGGACGGGTCGATGTCAC |
| *Runx1* | F | GATGGCACTCTGGTCACCG |
|  | R | GCCGCTCGGAAAAGGACAA |
| *Foxp3* | F | CACCCAGGAAAGACAGCAACC |
|  | R | GCAAGAGCTCTTGTCCATTGA |
| *Hif1a* | F | ACCTTCATCGGAAACTCCAAAG |
|  | R | CTGTTAGGCTGGGAAAAGTTAGG |
| *Cd14* | F | CTCTGTCCTTAAAGCGGCTTAC |
|  | R | GTTGCGGAGGTTCAAGATGTT |
| *Nfkb1* | F | ATGGCAGACGATGATCCCTAC |
|  | R | CGGAATCGAAATCCCCTCTGTT |
| *Stat1a* | F | CGGAGTCGGAGGCCCTAAT |
|  | R | ACAGCAGGTGCTTCTTAATGAG |
| *Crebbp* | F | TCTAGCATCAACCCAGGGATAG |
|  | R | GGCCATGTTTGTACTGTTCGG |
| *Calm3* | F | GATGGCACCATTACCACCAAG |
|  | R | CGCTGTCTGTATCCTTCATCTTT |
| *Cd3g* | F | ACTGTAGCCCAGACAAATAAAGC |
|  | R | TGCCCAGATTCCATGTGTTTT |
| *Tlr2* | F | CATCACCGGTCAGAAAACAA |
|  | R | GTCACCATGGCCAATGTAGG |
| *Il22* | F | CATGCAGGAGGTGGTACCTT |
|  | R | CAGACGCAAGCATTTCTCAG |
| *Muc2* | F | AGGGCTCGGAACTCCAGAAA |
|  | R | CCAGGGAATCGGTAGACATCG |
| *Muc4* | F | CCTCCTCTTGCTACCTGATGC |
|  | R | GGAACTTGGAGTATCCCTTGTTG |
| *Ido1* | F | CAAAGCAATCCCCACTGTATCC |
|  | R | ACAAAGTCACGCATCCTCTTAAA |
