# Supplementary material for: Antibiotic-induced acceleration of type 1 diabetes alters maturation of innate intestinal immunity
Source: eLife. 2018 Jul 25;7:e37816. doi: 10.7554/eLife.37816 (PMC6085123; doi:10.7554/eLife.37816)
Supplement: Transparent reporting form [file elife-37816-transrepform.docx]

***eLife’s* transparent reporting form**

We encourage authors to provide detailed information *within their submission* to facilitate the interpretation and replication of experiments. Authors can upload supporting documentation to indicate the use of appropriate reporting guidelines for health-related research (see [EQUATOR Network](http://www.equator-network.org/%20)), life science research (see the [BioSharing Information Resource](https://biosharing.org/)), or the [ARRIVE guidelines](http://www.plosbiology.org/article/info:doi/10.1371/journal.pbio.1000412) for reporting work involving animal research. Where applicable, authors should refer to any relevant reporting standards documents in this form.

If you have any questions, please consult our Journal Policies and/or contact us: [editorial@elifesciences.org](mailto:editorial@elifesciences.org).

**Sample-size estimation**

- You should state whether an appropriate sample size was computed when the study was being designed
- You should state the statistical method of sample size computation and any required assumptions
- If no explicit power analysis was used, you should describe how you decided what sample (replicate) size (number) to use

Please outline where this information can be found within the submission (e.g., sections or figure legends), or explain why this information doesn’t apply to your submission:

**Section: T1D development**

*T1D development:* There were 20-25 mice per group (total of 8 groups: 1PAT male; 1PAT-control male; 1PAT female; 1PAT control female; 3PAT male; 3PAT-control male; 3PAT female; 3PAT control female).

Kaplan-Meier analysis was performed for T1D development with the log-rank test to test for significance;

This information can be found in Figure 1B and Figure 1 legend;

*Insulitis:* There were 6 mice per group (2 groups: 1PAT; and control), with a total of 235 islets in the 1PAT group and 201 islets in the control group that were scored. Significance was determined using the chi square method.

This information can be found in the Figure 1 legend.

**Section: Microbiome**

*16S rRNA:* There were 20-25 fecal samples (total of 8 groups: 1PAT male; 1PAT-control male; 1PAT female; 1PAT control female; 3PAT male; 3PAT-control male; 3PAT female; 3PAT control female) at all 3 time points for the 16S rRNA high throughput sequencing analysis. The One-way-ANOVA method was used to assess the differences in α-diversity, and One-way-ANOVA with Tukey correction for multiple comparisons was used to assess the β-diversity differences. This information can be found in the Figure 2 legend.

*Metagenomics:* 6 fecal samples per group were tested for metagenomic sequencing BGC analysis. Fisher’s exact test with FDR correction method was used. This information can be found in the Figure 2 legend and in the Materials and Methods section “Microbiome assessment with whole genome shotgun sequencing”.

**Section: Metabolomics**

*SCFA:* There were 6-7 samples per group (2 groups: control and 1PAT) for cecal SCFA analysis. Welch’s T Test for unpaired samples was used. This information could be found in the Figure 3 legend.

*Serum and Liver*: There were 7-17 samples per group (2 groups: control and 1PAT) for both serum and liver untargeted metabolites analysis. Significant changes in pairwise comparisons were evaluated by Wilcoxon Rank-Sum test by SAS 9.4 (SAS Institute Inc., Cary NC). This information can be found in the Figure 3 legend and in the Materials and Methods section “GC-TOF-MS Metabolomics of liver and serum”.

**Section: Host gene expression**

*RNAseq and Nanostring:* There were 3-7 samples per group for RNAseq and Nanostring-based differential gene expression, and differential pathway analysis. Fisher’s exact test and Benjamini-Hochberg correction for correction of FDR were used. This information can be found in the Materials and Methods sections “RNA extraction and RNA-Seq”, “Immune gene NanoString analysis”, Figure 4 legend, and Figure 5 legend.

*RT-qPCR:* There were 6-22 samples per group for RT-qPCR-based gene expression analysis. The Mann Whitney test was used. This information can be found in the Figure 6 legend.

**Section: Histone modification analysis**

There were 4-5 samples per group. Welch’s t test was used. This information can be found in the Figure 7 legend and in the Materials and Methods section “Global histone modification analysis”.

**Section: Immune experiments**

*IgA ELISA:* There were 20-25 mice per group (4 groups: 1PAT male, 1PAT female, control male, and control female) at 3 timepoints for IgA ELISA analysis. The Mann Whitney test was used. This information can be found in the Figure 8 legend.

*Flow cytometry:* There were 10-12 samples per group (1PAT and control) for flow cytometric analysis. Unpaired T tests were used. This information can be found in the Figure 8 legend.

**Replicates**

- You should report how often each experiment was performed
- You should include a definition of biological versus technical replication
- The data obtained should be provided and sufficient information should be provided to indicate the number of independent biological and/or technical replicates
- If you encountered any outliers, you should describe how these were handled
- Criteria for exclusion/inclusion of data should be clearly stated
- High-throughput sequence data should be uploaded before submission, with a private link for reviewers provided (these are available from both GEO and ArrayExpress)

Please outline where this information can be found within the submission (e.g., sections or figure legends), or explain why this information doesn’t apply to your submission:

Our main experiment involved ~200 mice, in which we have continuously tracked T1D development in each mouse, and we collected tissue samples just once for analysis from each animal (n=20-25 animals for each of the 8 groups). In the second, nested-experiment we obtained samples from ~ 280 mice at sacrifice at intervals ranging from P2 to P70. The groups of mice at each of the time points was 2-4, including 6-14 mice for each group (PAT male, Control male, PAT female, Control female) from P12-P70, and 4 mice for each group (male and female) at P2 before PAT.

We have uploaded High-throughput sequencing data, as suggested, which has been indicated in the Materials and Methods section “Data deposition”. RNA-Seq data that support the findings of this study have been deposited in the ArrayExpress database (www.ebi.ac.uk/arrayexpress) with accession code E-MTAB-6826 (https://www.ebi.ac.uk/arrayexpress/experiments/E-MTAB-6826). 16S rRNA data have been deposited in QIITA (https://qiita.ucsd.edu/) (<https://qiita.ucsd.edu/study/description/11242>) under identifier 11242. Ileal NanoString data have been deposited in the NCBI Gene Expression Omnibus (https://www.ncbi.nlm.nih.gov/geo/) and are accessible through GEO Series accession GSE101721 (https://www.ncbi.nlm.nih.gov/geo/query/acc.cgi?acc=GSE10172).

Shotgun metagenomics data have been deposited in the European Nucleotide Archive (ENA) (https://www.ebi.ac.uk/metagenomics/) under accession number, PRJEB26585 (http://www.ebi.ac.uk/ena/data/view/PRJEB26585). Metabolomics data have been deposited at the NIH Common Fund Metabolomics Workbench ([www.metabolomicsworkbench.org](http://www.metabolomicsworkbench.org)) with doi: 10.21228/M8C39R. This information can be found in the Materials and Methods section “Data deposition”.

**Statistical reporting**

- Statistical analysis methods should be described and justified
- Raw data should be presented in figures whenever informative to do so (typically when N per group is less than 10)
- For each experiment, you should identify the statistical tests used, exact values of N, definitions of center, methods of multiple test correction, and dispersion and precision measures (e.g., mean, median, SD, SEM, confidence intervals; and, for the major substantive results, a measure of effect size (e.g., Pearson's r, Cohen's d)
- Report exact p-values wherever possible alongside the summary statistics and 95% confidence intervals. These should be reported for all key questions and not only when the p-value is less than 0.05.

Please outline where this information can be found within the submission (e.g., sections or figure legends), or explain why this information doesn’t apply to your submission:

For each experiment, we have included descriptions of the statistical analysis in the Methods sections and also in the Figure legends.

**Section: T1D development**

*T1D development:* Kaplan-Meier analysis for T1D development with the log-rank test for significance; exact p values are reported. This information could be found in Figure 1B and Figure 1 legend.

*Insulitis:* The Chi-squared method was used for comparing normal islets and islets with inflammation, and exact p values reported. This information can be found in the Figure 1 legend.

**Section: Microbiome**

*16S rRNA:* The One-way-ANOVA method was used to assess for differences in α-diversity, and One-way-ANOVA with Tukey correction for multiple comparisons was used to assess for differences in for β-diversity. Since many p values were determined and were highly significant, we report values with p<0.0001. This information can be found in the Figure 2 legend.

*Metagenomics:* Fisher’s exact test with FDR correction was used for the metagenomic sequencing BGC analysis. This information can be found in the Figure 2 legend and Materials and Methods section “Microbiome assessment with whole genome shotgun sequencing”.

**Section: Metabolomics**

*Untargeted metabolomes:* Significant changes in pairwise comparisons were evaluated by Wilcoxon Rank-Sum test for untargeted metabolomics analysis in the serum and liver samples. In Supplementary file 3, we report the exact p value for each identified metabolite. This information can be found in the Materials and Methods section “GC-TOF-MS Metabolomics of liver and serum”, and supplementary file 3.

*SCFA:* Welch’s T Test for unpaired samples was used for the analysis of cecal SCFAs. Mean values with STDEV are shown in Figure 3. This information can be found in the Figure 3 legend.

**Section: Host gene expression**

*RNAseq and Nanostring:* Fisher’s exact test and Benjamini-Hochberg FDR correction were used for the RNAseq and Nanostring-based assessments of differential gene expression, and pathway differential analysis. In the Supplemental data files, we report the exact p value or adjusted p value for each target gene. This information can be found in: Materials and Methods sections “RNA extraction and RNA-Seq” and “Immune gene NanoString analysis”, Figure 4 legend, and the Figure 5 legend.

*RT-qPCR:* The Mann Whitney test was used for RT-qPCR-based gene expression analysis. Mean values and STDEV are shown in Figure 6. This information can be found in the Figure 6 legend.

**Section: Histone modification analysis**

Welch’s t test was used. This information can be found in the Figure 7 legend and in the Materials and Methods section “Global histone modification analysis”. The number of significantly altered histone peptides in the male and female ileum or liver tissues are determined by Chi-squared analysis. This information can be found in the Figure 7 legend.

**Section: Immunologic experiments**

*IgA ELISA:* The Mann Whitney test was used to assess the IgA ELISA analysis. Mean values and STDEV are shown in the figure. This information could be found in the legend of Figure 8A.

*Flow cytometry:* Unpaired T tests were used for flow cytometric analysis. Mean values and STDEV are shown in the figure. This information can be found in the legend of Figure 8B.

(For large datasets, or papers with a very large number of statistical tests, you may upload a single table file with tests, Ns, etc., with reference to sections in the manuscript.)

**Group allocation**

- Indicate how samples were allocated into experimental groups (in the case of clinical studies, please specify allocation to treatment method); if randomization was used, please also state if restricted randomization was applied
- Indicate if masking was used during group allocation, data collection and/or data analysis

Please outline where this information can be found within the submission (e.g., sections or figure legends), or explain why this information doesn’t apply to your submission:

Mice were randomly grouped. This information can be found in the Materials and Methods section “Mice and antibiotic exposure”.

**Additional data files (“source data”)**

- We encourage you to upload relevant additional data files, such as numerical data that are represented as a graph in a figure, or as a summary table
- Where provided, these should be in the most useful format, and they can be uploaded as “Source data” files linked to a main figure or table
- Include model definition files including the full list of parameters used
- Include code used for data analysis (e.g., R, MatLab)
- Avoid stating that data files are “available upon request”

Please indicate the figures or tables for which source data files have been provided:

Our “relevant data” have been included in the Supplementary files, Source data files (including the Key Resource Table), or deposited in public databases online.
